# Comparative genomics and bioinformatics approaches revealed the role of CC-NBS-LRR genes under multiple stresses in passion fruit

**DOI:** 10.3389/fgene.2024.1358134

**Published:** 2024-02-26

**Authors:** Komal Zia, Muhammad Sadaqat, Baopeng Ding, Kinza Fatima, Norah A. Albekairi, Abdulrahman Alshammari, Muhammad Tahir ul Qamar

**Affiliations:** ^1^ Integrative Omics and Molecular Modeling Laboratory, Department of Bioinformatics and Biotechnology, Government College University Faisalabad (GCUF), Faisalabad, Pakistan; ^2^ College of Horticulture, Shanxi Agricultural University, Taigu, Shanxi, China; ^3^ Department of Pharmacology and Toxicology, College of Pharmacy, King Saud University, Riyadh, Saudi Arabia

**Keywords:** passion fruit, *CNL*, pathogen resistance, gene ontology, expression profiling, machine learning

## Abstract

Passion fruit is widely cultivated in tropical, subtropical regions of the world. The attack of bacterial and fungal diseases, and environmental factors heavily affect the yield and productivity of the passion fruit. The *CC-NBS-LRR* (CNL) gene family being a subclass of R-genes protects the plant against the attack of pathogens and plays a major role in effector-triggered immunity (ETI). However, no information is available regarding this gene family in passion fruit. To address the underlying problem a total of 25 and 21 *CNL* genes have been identified in the genome of purple (*Passiflora edulis* Sims.) and yellow (*Passiflora edulis f. flavicarpa*) passion fruit respectively. Phylogenetic tree was divided into four groups with PeCNLs present in 3 groups only. Gene structure analysis revealed that number of exons ranged from 1 to 9 with 1 being most common. Most of the *PeCNL* genes were clustered at the chromosome 3 and underwent strong purifying selection, expanded through segmental (17 gene pairs) and tandem duplications (17 gene pairs). *PeCNL* genes contained *cis-*elements involved in plant growth, hormones, and stress response. Transcriptome data indicated that *PeCNL3, PeCNL13,* and *PeCNL14* were found to be differentially expressed under Cucumber mosaic virus and cold stress. Three genes were validated to be multi-stress responsive by applying Random Forest model of machine learning. To comprehend the biological functions of PeCNL proteins, their 3D structure and gene ontology (GO) enrichment analysis were done. Our research analyzed the *CNL* gene family in passion fruit to understand stress regulation and improve resilience. This study lays the groundwork for future investigations aimed at enhancing the genetic composition of passion fruit to ensure robust growth and productivity in challenging environments.

## 1 Introduction

Fresh fruits are consumed all over the world as they are rich sources of vitamins and help boost the immune system to fight against diseases. Passion fruit (*P. edulis*) is also widely cultivated in countries across the globe due to its nutritional benefits and used in the production of juice, oil, jelly, etc. *P. edulis* belongs to the Passifloraceae family and is available in a variety of botanical forms including yellow passion fruit (*P. edulis* f. *flavicarpa*), water lemon (*Passiflora laurifolia*), purple passion fruit (*P. edulis* Sims.), fragrant granadilla (*Passiflora alata*), and others (Passiflora et al., 2021; Correia et al., 2022; Fonseca et al., 2022). A recent study involving comparative analysis of *P. edulis Sims.* and *P. edulis f. flavicarpa* demonstrated that the purple cultivar is more resistant to the pathogens than the yellow cultivar which highlights the importance of the purple cultivar (Rizwan et al., 2021). Apart from the uses of *P. edulis* in the food industry, it can also be useful for disease prevention due to the presence of antioxidants and phytochemicals in it. A well-known example in this regard is *Passiflora incarnata*, a plant with a well-established history in traditional herbal medicine, which has been utilized for its potential medicinal properties in alleviating hypertension, anxiety, and insomnia (Miroddi et al., 2013). Producers of passion fruit include Brazil, Asia, South Africa, and South America. The overall production of *P. edulis* gets reduced due to a variety of diseases including bacteriosis, anthracnose, fusarium wilt, and fruit woodiness which cause loss to the *P. edulis* producers (Joy P.P. and Sherin C.G, 1983; Xu et al., 2022).

**FIGURE 1:**
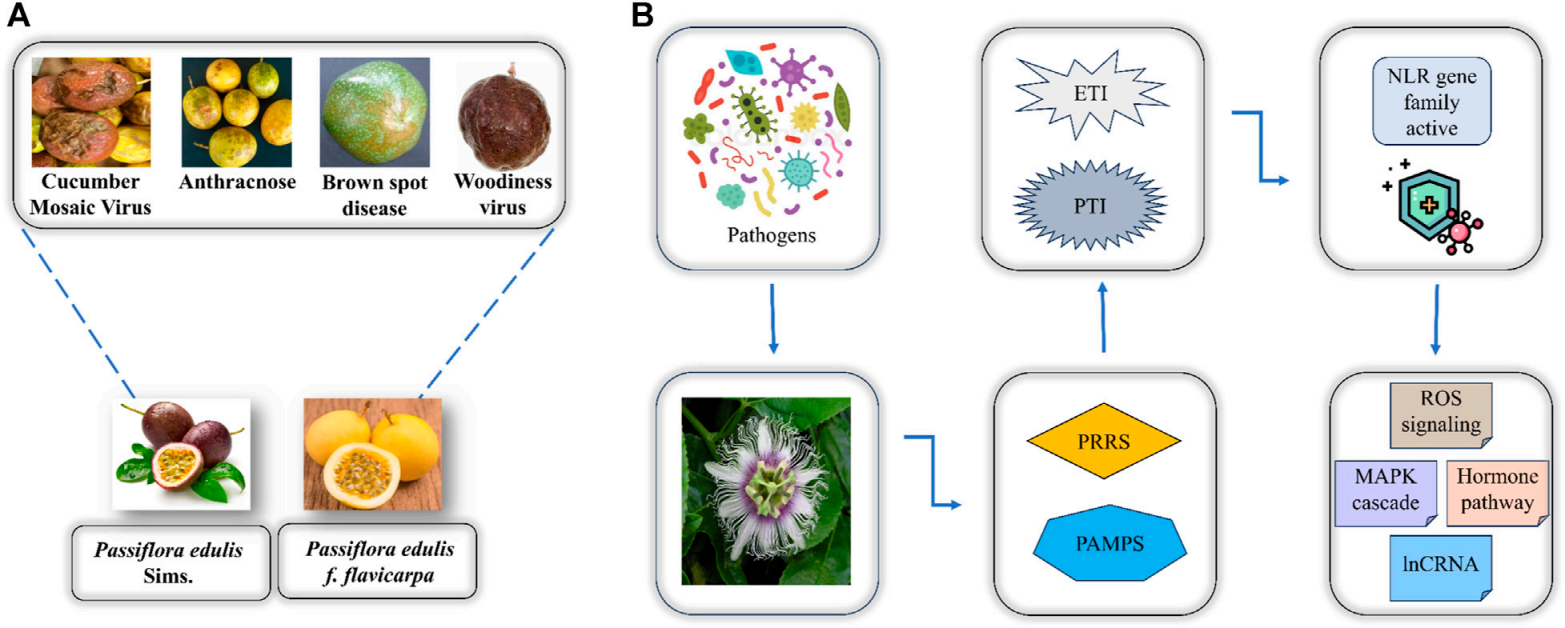
**(A)** Two major varieties of *P. edulis* and the associated diseases caused by the attack of pathogens. **(B)** Overview of how *P. edulis* responds to the attack of pathogens.

To better interpret the defense mechanism of *P. edulis* towards these diseases there is a need to identify disease resistance genes in this fruit. Two defense mechanisms are utilized by plants when they undergo pathogen stress including immunity activated by pathogen-associated molecular pattern (PTI) and effector-triggered immunity (ETI) (Delplace et al., 2022). The PTI involves the recognition of pathogens by specific pathogen recognition receptors (PRRs) at the cell membrane thereby inducing immunity in plants. However, the pathogens can release effectors as a contradictory effect to PTI thus leading to the activation of the ETI that protects plants by resisting the invasion of pathogens. When the former defense mechanism is unable to protect the plant from pathogen invasion then in later stages of plant immune response the effector-triggered immunity becomes active that is where the whole NBS-LRR (NLR) gene family has a crucial function i.e., CC-NBS-LRR (CNL) and Toll interleukin-NBS-LRR (TNL) act as sensors to the pathogenic effectors thereby initiating signaling mechanism where RPW8-NBS-LRR (RNL) function in assisting the plant resistance towards pathogen as depicted in (Figure 1) (Kaur et al., 2022).

The NLR gene family represents the most extensive group of R genes responsible for providing disease resistance in plants. This gene family is characterized by the presence of nucleotide binding site (NBS) and leucine-rich repeat (LRR) domains. This gene family has been classified into two main subfamilies including CC-NBS-LRR (CNL) and TIR-NBS-LRR (TNL) by the presence of coiled-coil and toll interleukin receptor domains at the protein’s N terminal region (Bezerra-Neto et al., 2019). Passion fruit holds significant economic, agricultural, industrial, and ornamental value. Owing to its multifaceted importance, addressing the challenges posed by pathogenic attacks and environmental stress becomes imperative to ensure sustained passion fruit yield and mitigate global reductions in fruit productivity (Chavarría-Perez et al., 2020; Rizwan et al., 2021). The modern era holds promise to improve the breeding strategies of plants by employing artificial intelligence and machine learning-based approaches to facilitate multi-omics data analysis eventually moving into the era of precision agriculture (Zhang et al., 2022). Once the R genes are identified in the genome of passion fruit, it will become easier to develop plants with improved resistance to pathogens and environmental stresses, eventually leading to increased productivity and yield (Gururani et al., 2012).

The CNL subclass has been previously reported in several plants including 51 members in *Arabidopsis thaliana* (Meyers et al., 2003), 159 members in *Oryza sativa* (Zhou et al., 2004), 119 members in *Populus trichocarpa* (Kohler et al., 2008)*,* 33 members *in Cucumis sativus* (Zhang et al., 2022)*,* 40 members in *Brassica rapa* (Y. Liu et al., 2021)*,* 361 in *Solanum tuberosum* (Jupe et al., 2012)*,* 78 in *Solanum pimpinellifolium* (Wei et al., 2020)*,* 14 in *Lagenaria siceraria,* 146 in *Triticum urartu* (Qian et al., 2021)*,* 166 in *Discorea rotundata,* 103 in *Glycine max* (Afzal et al., 2022), 467 in *Hordeum vulgare* (Liu et al., 2017), 54 in *Broussonetia papyrifera* (X. Zhang et al., 2023), 95 in *Elaeis guineensis* (Rosli et al., 2018)*,* 10 *in Citrus sinensis* (Yin et al., 2023), 47 in *Alphonso*, 27 in *Hong Xiang Ya*, and 36 in *Tommy atkins* (ul Qamar et al., 2023). Machine learning approaches have also been applied in studies reported previously for the elucidation of candidate genes implicated in multi-stress responsiveness in *Oryza sativa* and *Sorghum bicolor* (Woldesemayat et al., 2018; Ramkumar et al., 2022).

The identification of passion fruit CNLs sheds light on their role in plant defense mechanisms against environmental stresses. This study provides a comprehensive structural evaluation, encompassing gene structure, motif analysis, phylogenetics, chromosomal distribution, *cis*-elements, gene enrichment, and 3D structure prediction. Additionally, it investigates the differential expression of these genes under disease and cold conditions, identifying multi-stress-responsive genes. The involvement of these CNLs in multi-stress responsiveness is further validated using a machine learning classifier algorithm. This research significantly contributes to our understanding of the CNL gene family in passion fruit, highlighting their importance in conferring resistance against various environmental stresses. The insights gained from this study will be invaluable for future researchers in the field.

## 2 Methods

### 2.1 Identification and physiochemical characterization of *CNL* genes in *P. edulis* Sims*.* and *P. edulis f. flavicarpa*

The 51 CNL protein sequences of *A. thaliana* obtained from the Ensembl Plants database (https://plants.ensembl.org/index.html) were used as query sequences against *P. edulis* Sims*.* (https://ftp.cngb.org/pub/CNSA/data3/CNP0001287/CNS0275691/CNA0017758/). proteome database by utilizing the standalone version of BLASTp. The same 51 CNL protein sequences of *A. thaliana* were queried against the *P. edulis f. flavicarpa* proteome database by performing the BLASTp search at the Passion fruit genomic database (http://passionfruit.com.cn/). After manual verification, all the duplicates were removed and a list of all unique IDs was further processed. Subsequently, this list underwent further processing to check the presence of specific CNL domains, namely, the coiled-coil (CC) domain, NB-ARC domain, and LRR domain, utilizing the Pfam (https://pfam-legacy.xfam.org) (Mistry et al., 2021) CDD (https://www.ncbi.nlm.nih.gov/Structure/bwrpsb/bwrpsb.cgi) (Lu et al., 2020), HMMER (https://www.ebi.ac.uk/Tools/hmmer/search/hmmscan) (Potter et al., 2018), and Interpro (https://www.ebi.ac.uk/interpro/search/sequence/) (Hunter et al., 2009) databases for domain identification. The presence of the coiled-coil domain was validated through Paircoil2 (http://cb.csail.mit.edu/cb/paircoil2/paircoil2.html) (McDonnell et al., 2006). The IDs were selected for further analysis that contained CNL-specific domains.

To gain a more profound understanding of the properties of the identified PeCNL proteins, their length (aa), molecular weight (MW), isoelectric point (pI), aliphatic index (AI), and grand average of hydropathicity (GRAVY) values were calculated using EXPASY ProtParam tool (https://web.expasy.org/protparam/) (Artimo et al., 2012). The Plant-mPLoc web server (http://www.csbio.sjtu.edu.cn/cgi-bin/PlantmPLoc.cgi) (Chou and Shen, 2010) has been utilized to identify the subcellular location of PeCNL proteins.

### 2.2 Multiple sequence alignment and phylogenetic analysis

The multiple sequence alignment of the underlying protein sequences of *P. edulis* Sims*.* (25), *A. thaliana* (51), *M. domestica* (21), *C. sativus* (33), and *B. oleracea* (33) that belonged to CNL subclass were submitted to ClustalW at MEGA 7.0 software to identify the highly conserved amino acid residues (Kumar et al., 2016). To infer the evolutionary relationships of CNL proteins of *P. edulis* with other plants the aligned sequences were subjected to construct a phylogenetic tree based on the Neighbor-Joining (NJ) method with 1000 bootstrap using PAUP4 software (Wilgenbusch and Swofford, 2003), and iTOL V6 was utilized for the editing of the phylogenetic tree (https://itol.embl.de) (Letunic and Bork, 2021).

### 2.3 Conserved motifs and gene structures

The complete and accurate representation of genetic structures of identified *PeCNL* genes will be demonstrated by utilizing the CDS and gene sequences of *P. edulis* Sims. The CDS and gene sequences were retrieved from the CNSA resource. The retrieved sequences were submitted to the Gene Structure Display Server 2.0 (GSDS; https://gsds.gao-lab.org) web server (Hu et al., 2015) for visualizing the gene structures. The prediction of highly conserved motifs associated with the proper functioning of the PeCNL proteins, the protein sequences were submitted to MEME suite 5.4.1 (https://meme-suite.org/meme/tools/meme) (Bailey et al., 2009), with the maximum number of motifs set to 10 and the other parameters were set to the default.

### 2.4 Analysis of gene location, gene duplication, and *cis*-regulatory elements (CREs)

To check the tendency of how well the CNL genes tend to cluster together at the respective chromosomes, genes were mapped to their respective positions at chromosomes. The information related to chromosome number and position of each *PeCNL* gene was acquired by using the annotation file (.gff3) of *P. edulis* Sims. downloaded from the CNSA database (https://ftp.cngb.org/pub/CNSA/data3/CNP0001287/CNS0275691/CNA0017758/) (Guo et al., 2020). To visualize the distribution patterns of *PeCNL* genes at chromosomes TBtools software v1.116 (Chen et al., 2020) has been utilized. Also to get insights into the duplication type and its impact on the evolution gene duplication analysis has been conducted. Among all the identified *PeCNL* genes, the sequences that shared the sequence identity of ≥70% were considered to be duplicates. DnaSP v6 software (Librado and Rozas, 2009) was used to calculate the rate of both synonymous (Ks) and non-synonymous (Ka) substitutions. The Ka/Ks ratio was used to demonstrate the selection pressure that aided in the evolution of the CNL gene family in *P. edulis* Sims. Duplication time was calculated based on the formula: T = Ks/2x (where x = 6.56 × 10^−9^ for dicots) (Zameer et al., 2022; Zia et al., 2022; Sadaqat et al., 2023).

To decipher the transcription factors and their associated functions in the identified genes promoter regions were analyzed to find the appropriate *cis*-element present in each gene. The 1000bp upstream promoter sequences of the identified *PeCNL* genes were retrieved via TBtools v1.116 and submitted to the PlantCare database (https://bioinformatics.psb.ugent.be/webtools/plantcare/html/) (Lescot et al., 2002) to find the potential *cis*-regulatory elements.

### 2.5 Protein-protein interaction (PPI) and miRNA target prediction

To check the interaction of PeCNL proteins with the interactions reported earlier in other plants the identified PeCNL protein sequences were subjected to the STRING database (https://string-db.org) (Szklarczyk et al., 2021). miRNAs were predicted to be able to particularly control the expression patterns of CNL genes after experimental validation because to silence or increase the gene expression the corresponding miRNA can be targeted in each case. A list of predicted miRNAs for *P. edulis* was downloaded from the already reported study (Paul et al., 2020) and the psRNATarget web server (https://www.zhaolab.org/psRNATarget/) (Dai et al., 2018) was utilized to determine how these miRNAs were regulating the expression of the target *PeCNL* genes. The miRNA target gene network and PPI network were visualized by Cytoscape v3.8.2 (Paul Shannon et al., 1971).

### 2.6 Expression profiling of *PeCNL* genes under multiple stresses

To elucidate the expression patterns of *P. edulis* under the influence of Cucumber Mosaic Virus (CMV) infection and cold stress conditions, RNA-seq data were retrieved from the NCBI-SRA database under BioProject: PRJNA633743 and PRJNA634206 respectively. The genome (.fa) and annotation files (.gff3) of *P. edulis* Sims. were retrieved from the CNSA database (Guo et al., 2020). Cleaned paired-end reads were aligned to the reference genome by using a fast and sensitive alignment tool HISAT2 (Wen, 2017). To quantify the expression of *PeCNL* genes, Featurecounts (Liao et al., 2014) were used. Based on count values circular heatmaps were generated to visually represent the differential expression patterns of genes through chiplot (https://www.chiplot.online). The process of analyzing expression patterns of each PeCNL gene will be increasingly helpful in identifying PeCNL genes that are differentially expressed in various stresses.

### 2.7 Validation of *PeCNLs* under multiple stresses via machine learning

To explore the potential impact of *PeCNL* genes under multiple stresses machine learning approaches have been applied to the cold and CMV stress dataset of *P. edulis*. Cleaned reads were subjected to HISAT2 for aligning reads to the reference genome. To obtain the counts dataset of *PeCNL* genes under both stress conditions Featurecounts was utilized. Then DESeq2 (Michael et al., 2023) was applied to analyze the differentially expressed genes and to normalize the read counts. A Random Forest classifier (Chaudhary et al., 2016) was trained over counts data under CMV conditions. Then a threshold of logFC >0.05 and Padj. Val <0.05 was specified for upregulated genes and logFC < −0.05 and Padj.val <0.05 was selected for downregulated genes to identify common genes in each case. The Common genes were then used to test the model performance in terms of accuracy, sensitivity, and specificity towards predicting the multi-stress responsive genes.

### 2.8 3D structure prediction and gene ontology (GO) enrichment analysis of PeCNL proteins

To get detailed information regarding the structural conformation of the multi-stress related proteins their 3d structures have been predicted. Analyzing the impact of expression patterns of PeCNL genes in CMV-infected condition and cold only those proteins were selected for 3D structure prediction that were responsible for multi-stress responsiveness. Protein sequences of PeCNL3, PeCNL13, and PeCNL14 were submitted to the trRosseta web server (https://yanglab.nankai.edu.cn/trRosetta/) (Du et al., 2021). For validation of the predicted structures of selected PeCNL proteins, the SAVES server (https://saves.mbi.ucla.edu) was utilized to select model with the most favorable structure conformation and stability. To visualize the predicted 3D structures, PyMOL software was used (Yuan et al., 2017). To comprehend the biological function of the PeCNLs, the GO analysis was done by using the Pannzer2 database (http://ekhidna2.biocenter.helsinki.fi/sanspanz/) (Törönen et al., 2018). The GO has been classified into three categories: Biological Processes (BP), Cellular Components (CCs), and Molecular Functions (MF).

## 3 Results

### 3.1 Identification and physiochemical characterization of *CNL* genes in *P. edulis* Sims and *P. edulis f. flavicarpa*

**FIGURE 2:**
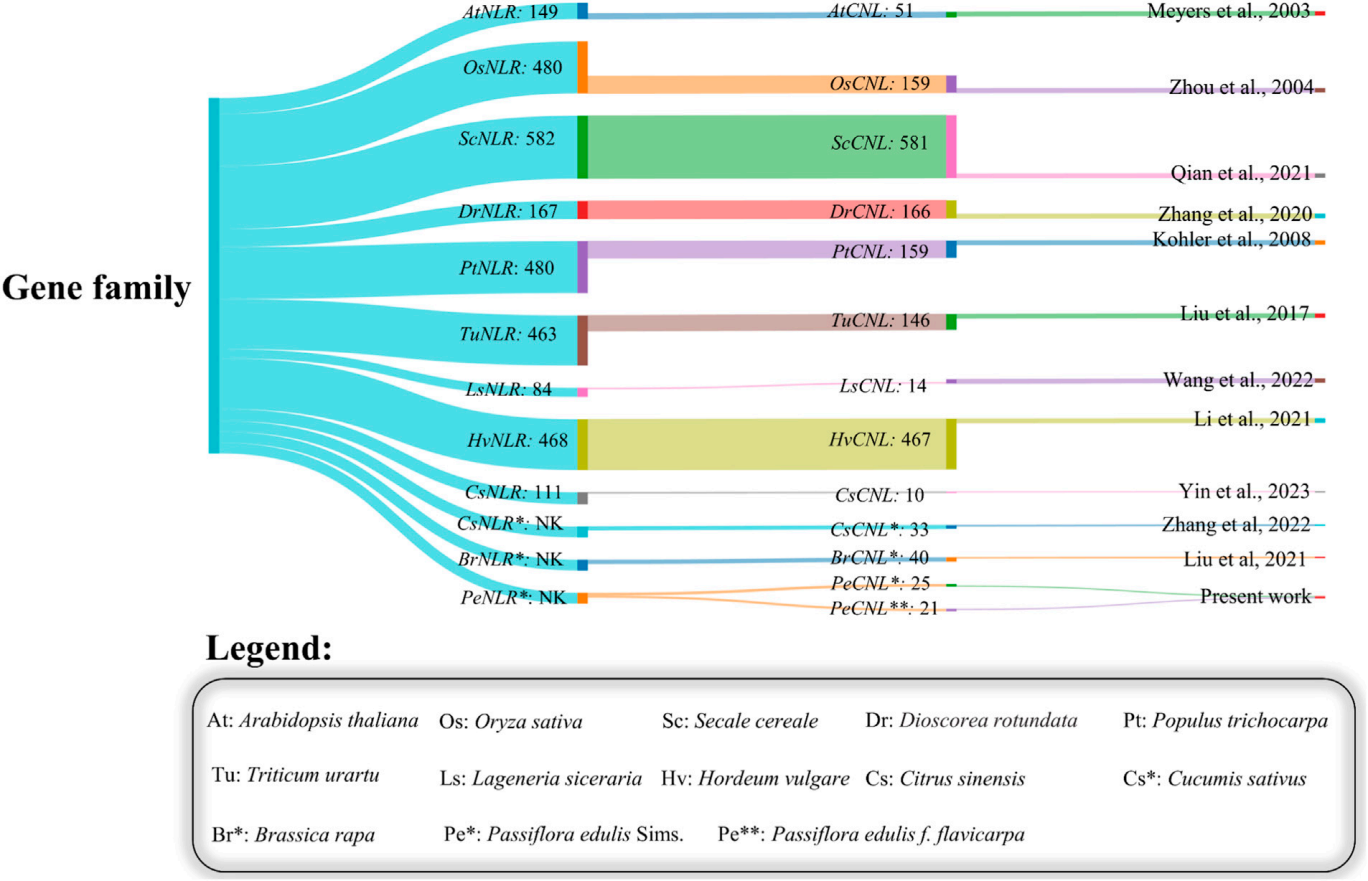
Sankey plot representing the variability in number of members identified in the NLR gene family and the number of members in the CNL gene family across different plants.

The presence of CNL-specific domains resulted in the successful identification of 25 *PeCNL* genes in *Passiflora edulis* Sims and 21 *PeCNL* genes in *Passiflora edulis f. flavicarpa*. The identified *CNL* genes in *P. edulis* Sims have been named according to the order in which they are present at chromosomes. The specific information regarding the properties of PeCNL proteins is given in (Supplementary Table S1 **)**. The conserved domains found in these proteins include Rx_N, NB-ARC, LRR_8, and RPW8. All the PeCNL proteins were predicted to have a CC domain. Most of the proteins contained Rx_N, NB-ARC, and LRR_8 domains. While the RPW8 domain was present only in PeCNL3. All the predicted domains were involved in disease resistance in *P. edulis* and other plants as mentioned in previous studies (Figure 2).

**FIGURE 3:**
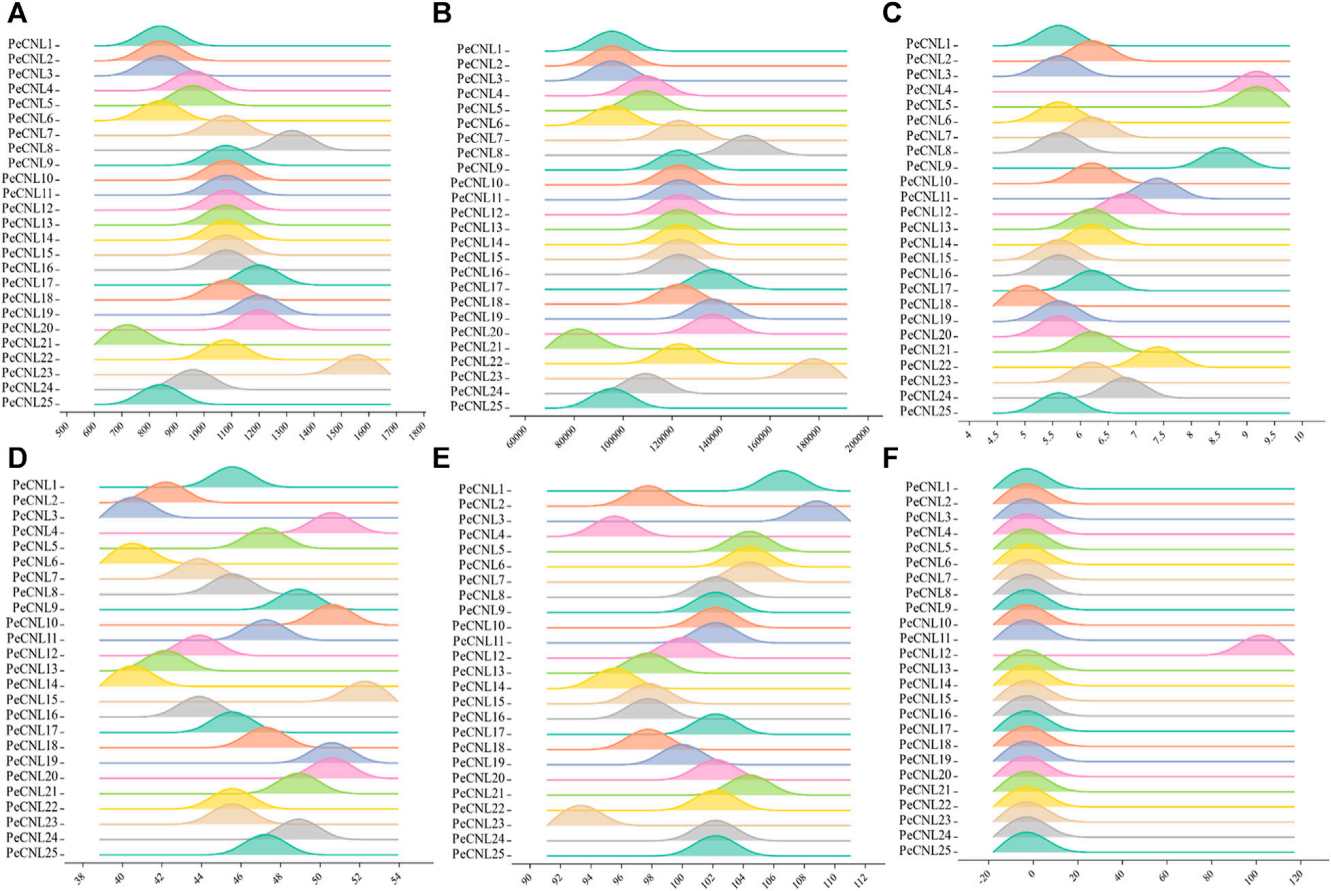
Visual representation of PeCNL proteins in *Passiflora edulis* Sims. calculated by Expasy Protparam server. **(A)** Length of PeCNL proteins **(B)** Molecular weight of PeCNL proteins*,* **(C)** Isoelectric point of PeCNLs, **(D)** Instability index of PeCNL proteins, **(E)** Aliphatic index of PeCNL proteins, **(F)** GRAVY value for PeCNL proteins.

The length of PeCNL proteins ranges from 741 to 1541 aa, while their molecular weight (MW) ranges from 84156.4 to 175592 (Da). The majority of the PeCNL proteins were acidic and only a few were basic according to the isoelectric points i.e., 5.12 to 9.09. All of the identified PeCNL proteins were unstable because the instability index was found to be greater than 40. The GRAVY value was negative for 24 PeCNL proteins suggesting that these were hydrophilic while only PeCNL12 had a GRAVY value positive meaning that it was hydrophobic (Figure 3). The proteins that are present outside the cell membrane or at the cell surface are always hydrophobic while the proteins that are present inside the cell are hydrophilic.

**FIGURE 4:**
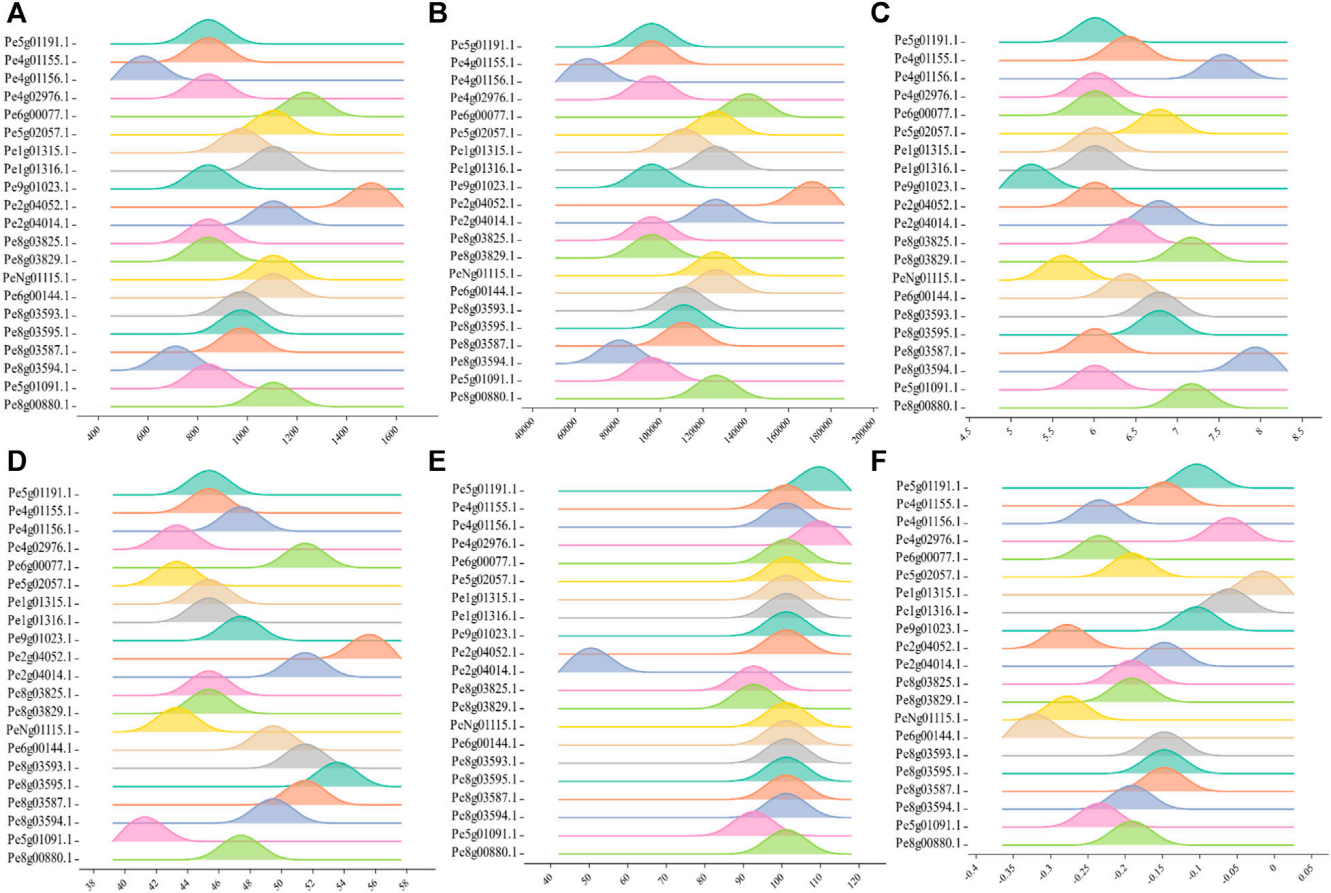
Graphical representation of physical and chemical properties of PeCNL proteins in *Passiflora edulis f. flavicarpa* calculated by Expasy Protparam. **(A)** Length of PeCNL proteins **(B)** Molecular weight of PeCNL proteins, **(C)** Isoelectric point of PeCNLs, **(D)** Instability index of PeCNL proteins, **(E)** Aliphatic index of PeCNL proteins, **(F)** GRAVY value for PeCNL proteins.

Most of the PeCNL proteins were predicted to be localized in the cytoplasm while some of the proteins were localized in the cell membrane (Supplementary Table S2). The conserved domains were the same in both cultivars while the properties of PeCNL proteins were variable in both cultivars and are given as follows. Characteristics for the yellow cultivar proteins were as follows. The length of PeCNL proteins ranged from 604 to 1478 (aa). PeCNL proteins have their molecular weights in the range from 90673 to 168831 (Da). Only 4 PeCNL proteins were basic and all of the remaining proteins were acidic, while all the proteins were unstable and hydrophilic (Figure 4). PeCNL proteins were predicted to be present in the cell membrane and cytoplasmic sections inside the cell.

### 3.2 Multiple sequence alignment and phylogenetic analysis

**FIGURE 5:**
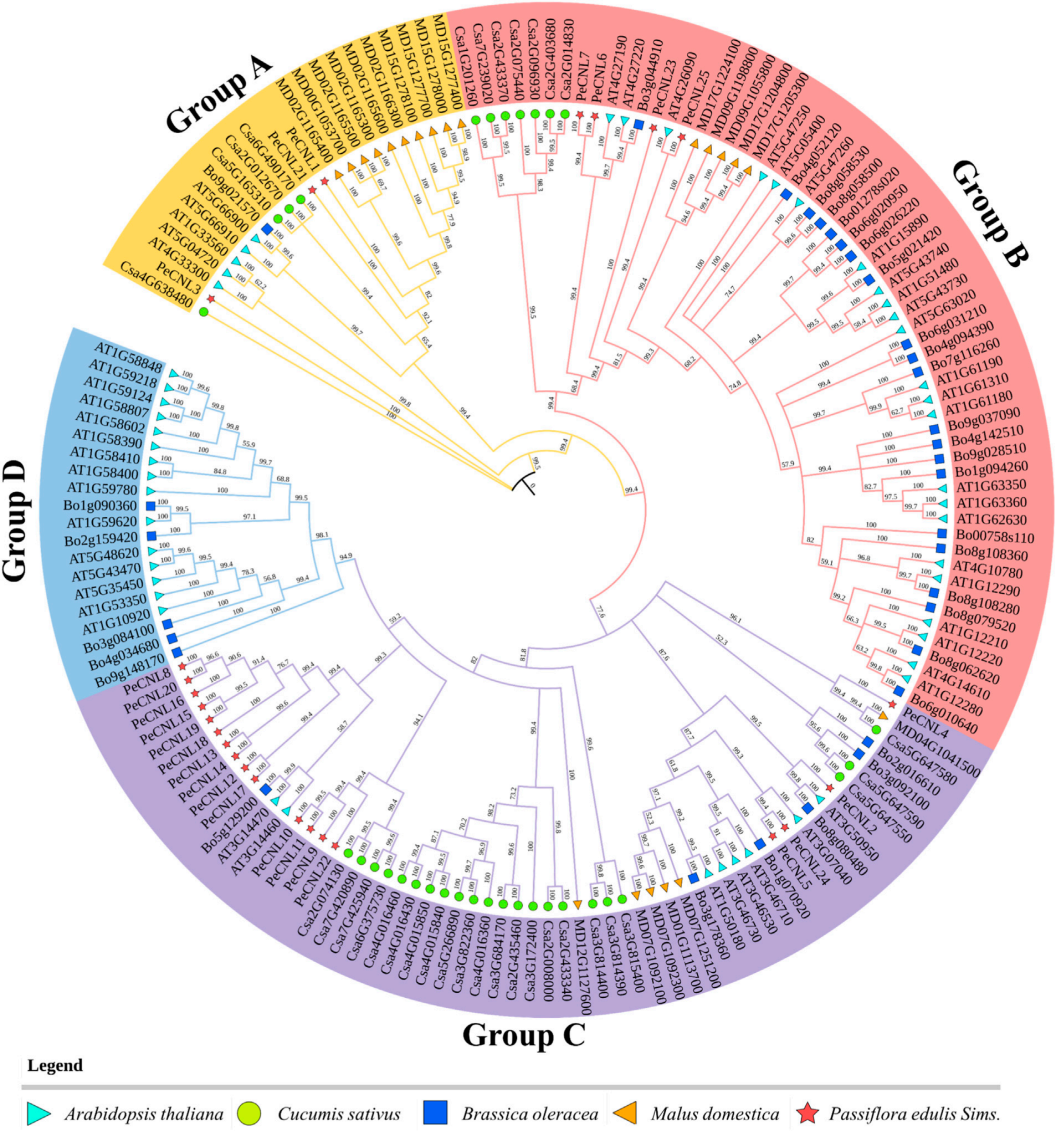
A phylogenetic tree encompassing CNL proteins from diverse plant species, including *A. thaliana, P. edulis* Sims., *C. sativus, B. oleracea,* and *M. domestica* was constructed. The tree was constructed using PAUP4 software relying on Neighbor-Joining (NJ) method, with 1000 bootstraps replicates. Each distinct group in the phylogenetic tree is represented by different colors.

To analyze the evolutionary relationships of PeCNL proteins with other plants, the aligned protein sequences of *A. thaliana, C. sativus, B. oleracea, P. edulis,* and *M. domestica* were subjected to phylogenetic analysis. The resultant tree was divided into four groups namely, A to D. All of the PeCNL proteins were present in groups A to C while none of the PeCNL protein was present in group D (Figure 5). Group A contained 23 members (3 PeCNLs, 4 CsCNLs, 1 BoCNL, 5 AtCNLs, and 10 MdCNLs), group B contained 60 members (4 PeCNLs, 7 CsCNLs, 21 BoCNLs, 23 AtCNLs, and 5 MdCNLs), group C contained 60 members (18 PeCNLs, 22 CsCNLs, 6 BoCNLs, 8 AtCNLs, and 6 MdCNLs) and group D contained least number of members i.e., 20 members (5 BoCNLs and 15 AtCNLs). The distribution of members in each group was consistent with those in *AtCNLs, CsCNLs,* and *BoCNLs* indicating that similar evolution patterns were shared by other plants. A monophyletic clade was formed for all plants present in group B indicating that all members of a monophyletic clade share a common evolutionary history and are more closely related to each other than they are to any other group of organisms. Due to the presence of the same conserved domains in other plants, it can be inferred that they could be involved in similar functions in each plant given the mode of evolution was different i.e., some of the members could be the products of speciation event giving rise to orthologs while others could be the products of duplication event i.e., paralogs but each of them shared close homology. Based on the phylogenetic tree it can be inferred that *P. edulis* shared close evolutionary relationships with *M. domestica* suggesting that they share a common ancestor. Besides, the AtCNL members were close orthologs of PeCNL members.

### 3.3 Conserved motifs and gene structures

**FIGURE 6:**
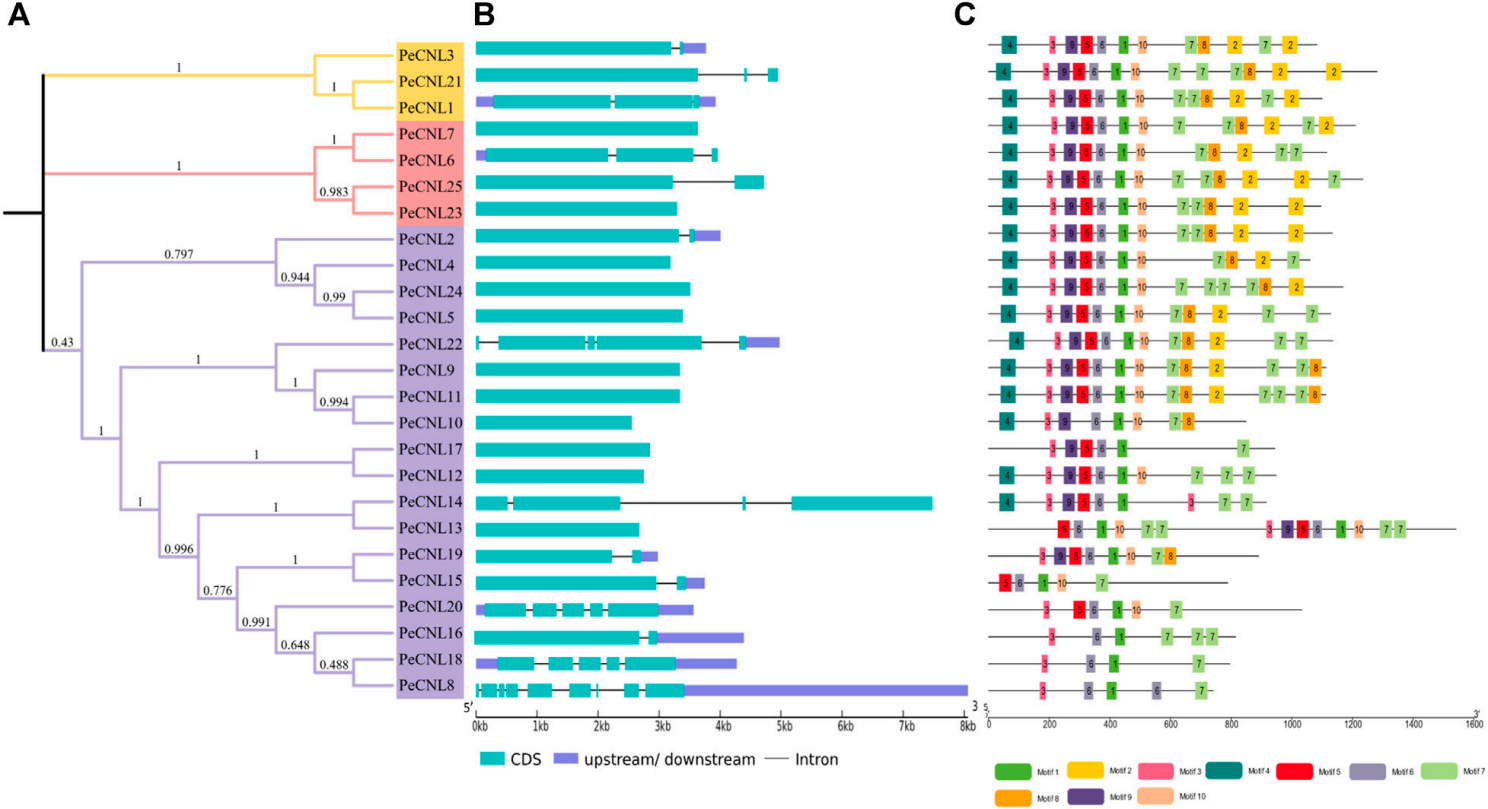
**(A)** For visualization of phylogenetic tree of PeCNL proteins iTOL was utilized. **(B)** Gene structure of *PeCNL* genes constructed by GSDS2.0., and **(C)** Conserved motifs in PeCNL proteins that have been predicted by using MEME suite 5.4.1.

The exon-intron patterns were roughly the same for nearly all genes as *PeCNL21* contained 9 exons and 8 introns and *PeCNL1*, *PeCNL3*, and *PeCNL9* had 5 exons and 4 introns. The number of exons in the remaining genes varied from 1 to 4, with 1 being the most prevalent among them. No intron was present in *PeCNL* genes with only one exon, and for the others, the number of introns ranged from 1 to 3 (Figures 6A, B).

A total of 10 conserved motifs that were predicted to be present in PeCNL proteins including motif 1 that represented CNBS-1 and RNBS-D motifs, motif 3 represented the P-loop, motif 5 represented the RNBS-B motif, motif 6 represented the GLPL motif, and motif 9 represented the kinase-2 motif (Figure 6C). The conserved motifs associated with proper functioning of CNL proteins have been conserved in all three subgroups except PeCNL8, PeCNL15, PeCNL16, PeCNL18, and PeCNL20 that lack motif 9 i.e., kinase-2 and other conserved motifs responsible for unknown functions.

Motifs 1, 3, 5, 6, and 9 represent the motifs particularly responsible are crucial for the structural confirmation and functioning of CNL proteins. These motifs are conserved across all three sub-groups, except for motif 9, which is absent in some proteins, such as PeCNL8, PeCNL15, PeCNL16, PeCNL18, and PeCNL20 of group 3, possibly due to diversity in conservation patterns. Motifs often play crucial roles in protein folding, stability, and interactions with other molecules. The absence of motif 4 in Subgroup 3 may suggest indicate a distinct functional specialization or structural variation within this subgroup (Figure 6C).

### 3.4 Analysis of gene location, gene duplication, and *cis-*regulatory elements (CREs)

**FIGURE 7:**
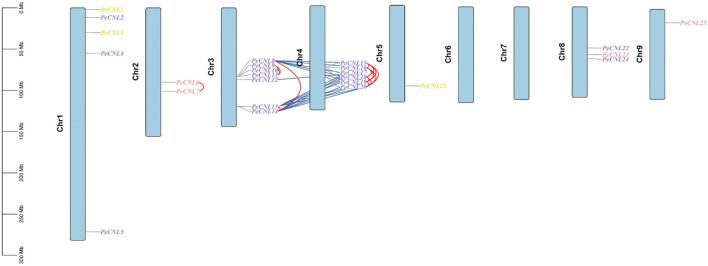
Distribution of 25 *PeCNL* genes at chromosomes based on their respective location. The vertical bar at left represents the size of chromosomes in Megabases. Tandem duplicates are indicated by red lines and segmental duplicates are indicated by dark blue colored lines. Different colors are used to represent the groups to which each gene belongs in the phylogenetic tree.

*PeCNL* genes followed uneven distribution patterns at 7 chromosomes. Among the 25 *PeCNL* genes identified none of the genes was present at chromosomes 6 and 7. Chromosome 5 and Chromosome 9 contained only one gene each namely, *PeCNL21* and *PeCNL25* and chromosome 3 had 7 genes present in the form of a cluster *PeCNL8*, *PeCNL9*, *PeCNL10*, *PeCNL11*, *PeCNL12*, *PeCNL13*, and *PeCNL14* (Figure 7). Besides, there were different numbers of genes present on each chromosome including 5 at chromosome 1, 2 at chromosome 2, 6 at chromosome 4, and 3 at chromosome 8.

A total of 34 duplicated gene pairs have been identified as a result of gene duplication analysis (Supplementary Table S3). 17 gene pairs have been segmentally duplicated including *PeCNL8/PeCNL15*, *PeCNL8/PeCNL16*, *PeCNL8/PeCNL18*, *PeCNL8/PeCNL19*, *PeCNL8/PeCNL20*, *PeCNL12/PeCNL17*, *PeCNL13/PeCNL15*, *PeCNL13/PeCNL16*, *PeCNL13/PeCNL17*, *PeCNL13/PeCNL19*, *PeCNL13/PeCNL20*, *PeCNL14/PeCNL15*, *PeCNL14/PeCNL16*, *PeCNL14/PeCNL17*, *PeCNL14/PeCNL18*, *PeCNL14/PeCNL19*, *PeCNL14/PeCNL20* while 17 gene pairs have been tandemly duplicated including *PeCNL6/PeCNL7*, *PeCNL8/PeCNL13*, *PeCNL8/PeCNL14*, *PeCNL9/PeCNL10*, *PeCNL9/PeCNL11*, *PeCNL10/PeCNL11*, *PeCNL13/PeCNL14*, *PeCNL15/PeCNL16*, *PeCNL15/PeCNL18*, *PeCNL15/PeCNL19*, *PeCNL15/PeCNL20*, *PeCNL16/PeCNL18*, *PeCNL16/PeCNL19*, *PeCNL16/PeCNL20*, *PeCNL18/PeCNL19*, *PeCNL18/PeCNL20*, *PeCNL19/PeCNL20*. It was noteworthy that among 34 duplicated gene pairs 33 gene pairs emerged as a product of purifying selection and only 1 gene pair *PeCNL8/PeCNL18* was the product of positive selection as the Ka/Ks ratio was <1 for the former duplicated gene pairs and >1 for the later gene pair. The divergence time taken by genes to duplicate has ranged from 0.77 MYA to 51.09 MYA. Thus, segmental and tandem duplications have collectively contributed to the evolutionary dynamics of the *PeCNL* genes in *P. edulis* Sims.

**FIGURE 8:**
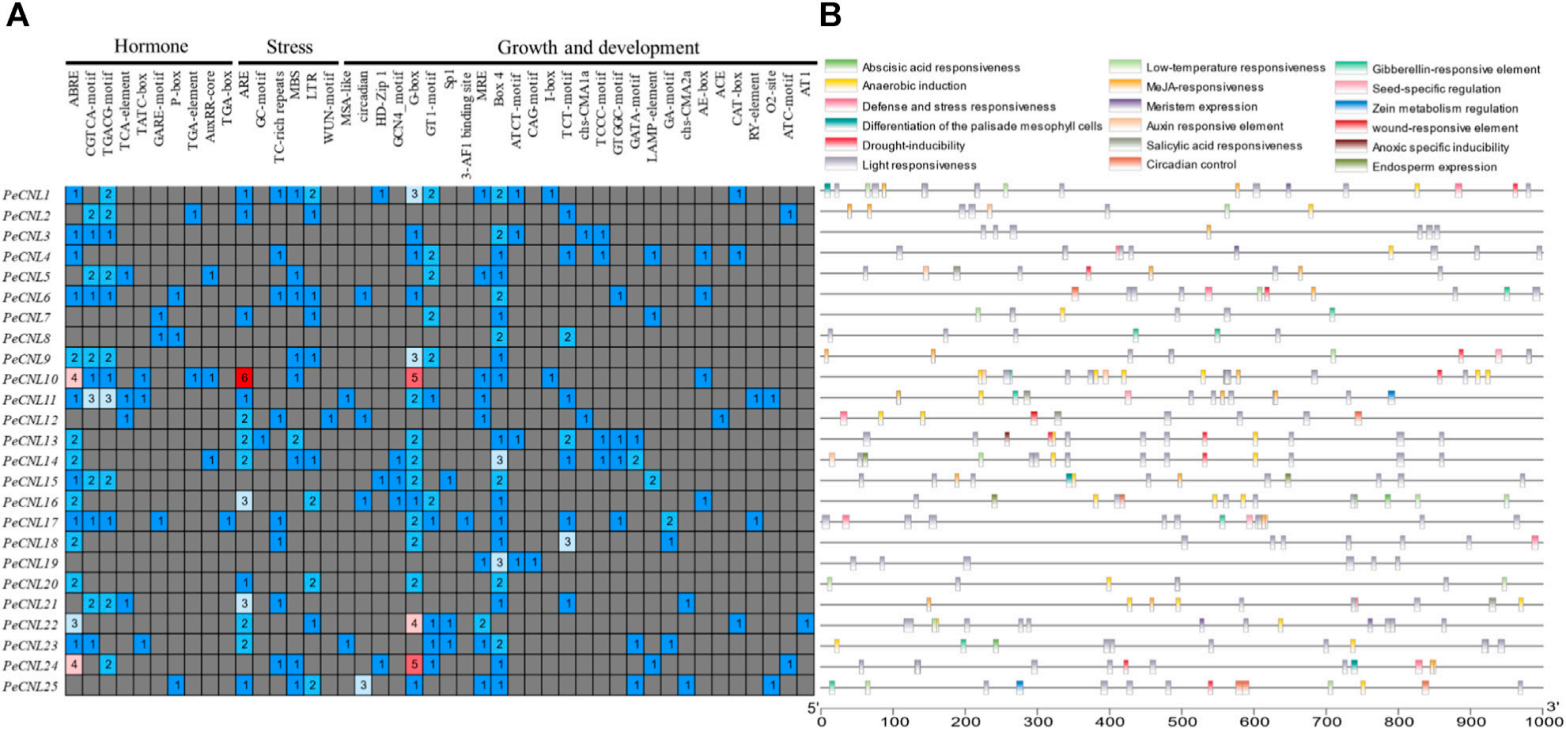
**(A)** Different categories for *cis-*elements present in promoter sequences of *PeCNL* genes. **(B)** Location of *cis*-element on each *PeCNL* gene.

Three different types of *cis-*regulatory elements were found in the promoter regions of *PeCNL* genes namely hormone-related, growth-related, and defense and stress-related. Hormone-related *cis*-elements belong to 10 different categories, growth-related *cis*-elements belong to 28 different types, and defense and stress-related *cis*-elements belong to 6 different types. Hormone-responsive *cis*-elements entailed following names AuxRR-core, TATC-box, ABRE, TGA-element, GARE-motif, P-box, CGTCA-motif, TGACG-motif, TCA-element, and TGA-box were involved in auxin, abscisic acid, methyl jasmonate, salicylic acid and gibberellin responsiveness. It is believed that hormone related *cis-*elements are responsible for pathogen induced immune response by mediating multiple signaling pathways. Hormone-related *cis*-elements, such as salicylic acid, jasmonic acid, and ethylene, along with other cis-elements like AS-1, G-box, GCC-box, and H-box, contribute to pathogen-induced immune responses in various plant species, enhancing resistance to pathogen attacks through signal transduction pathways activation. Growth and development-related *cis*-elements include light responsive, meristem expression, circadian control, endosperm expression, seed-specific regulation, zein metabolism regulation, differentiation of palisade mesophyll cells, anaerobic induction, and anoxic specific inducibility. Defense and stress-related *cis*-elements include low-temperature responsiveness, drought responsiveness, wound responsive element, and defense-related *cis*-element. Thus, CRE analysis revealed that the *PeCNL* genes are involved in the defense mechanism of *P. edulis* Sims. against a variety of pathogens and environmental stresses. The presence of TC-rich repeats, WUN-motif, ARE, GC-motif, LTR, and MBS has provided evidence for their involvement in the defense-related mechanism (Figure 8; Supplementary Table S4).

### 3.5 PPI and miRNA target prediction

**FIGURE 9:**
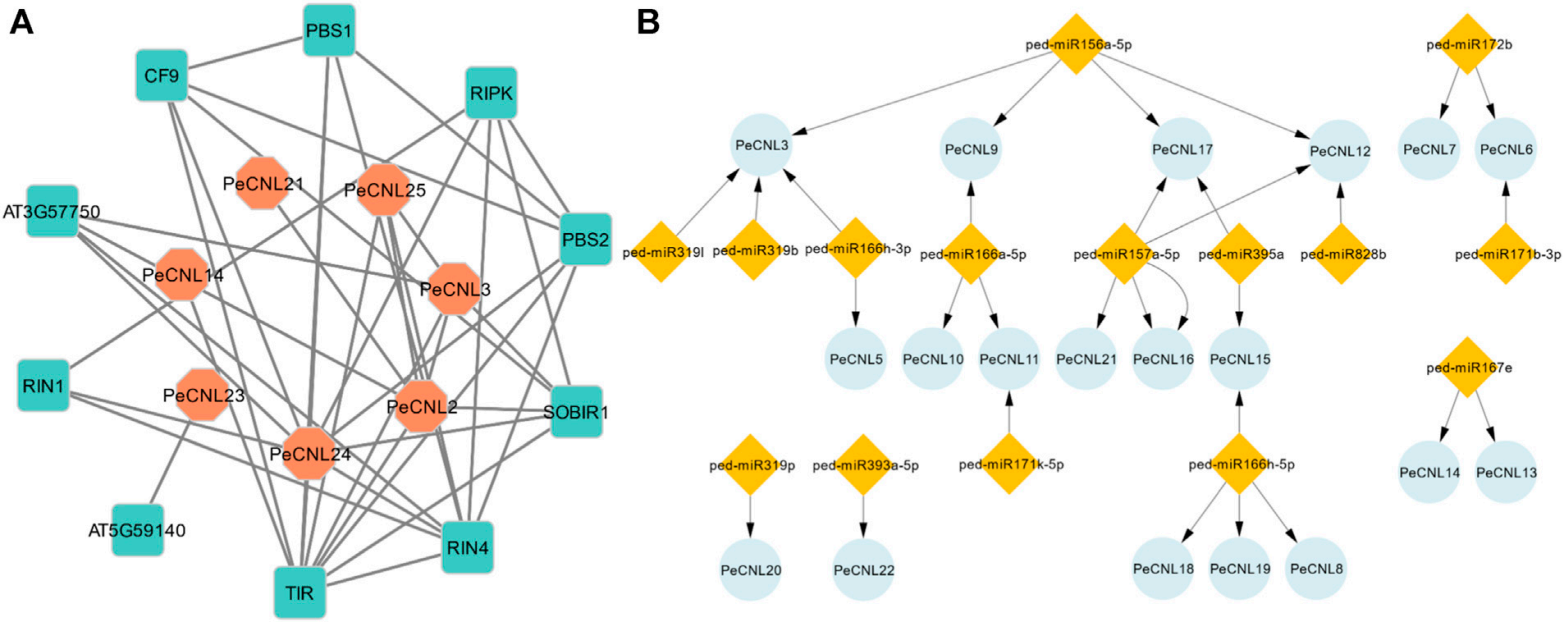
**(A)** Protein-Protein interaction network of PeCNL proteins with of *A. thaliana*’s proteins made by STRING database. **(B)** miRNA target gene network where the number of miRNAs that target each gene varies.

The interaction network was visualized at the second level of connection of PeCNL and other proteins. Among the identified potentially interacting proteins, the Toll/interleukin-1 receptor (TIR) exhibited the most significant degree of interaction, while PeCNL24 displayed the second-highest degree of connectivity. The interaction network consisted of seven proteins from *P. edulis* Sims, namely, PeCNL2, PeCNL3, PeCNL14, PeCNL21, PeCNL23, PeCNL24, and PeCNL25, that were interacting with 10 proteins of *A. thaliana*. (Figure 9A).

The Toll interleukin 1 protein of *A. thaliana* belongs to the TNL subclass of the NLR gene family and plays a significant role in the plant’s disease resistance mechanism. Additionally, the PeCNL24 protein is also involved in the plant’s disease resistance mechanism by recognizing RIN4 and conferring disease resistance. RIPK, RIN4, RIN1, PeCNL24, PeCNL25, SOBIR1, PeCNL3, AT3G57750, and PeCNL2 proteins were interacting with disease-resistant proteins in *A. thaliana*, and their mode of interaction was experimentally validated. Conversely, the remaining interactions were established through text-mining or other methods, yet their specific interactions have not been characterized experimentally.

A total of 15 miRNAs were found targeting 19 *PeCNL* genes having regulatory association with these miRNAs. Four miRNAs were targeting *PeCNL3* and *PeCNL12*, whereas three miRNAs were targeting *PeCNL17* respectively. The *PeCNL6, PeCNL9, PeCNL11,* and *PeCNL15* were targeted by two miRNAs while all of the remaining *PeCNL* genes were targeted by only one miRNA (Figure 9B; Supplementary Table S5). *PeCNL1*, *PeCNL2*, *PeCNL4*, *PeCNL23*, *PeCNL24*, and *PeCNL25* were not targeted by any of the miRNAs. The expectation score exhibited a range of 3.5 to 5. The prevailing function of most miRNAs is the inhibition of target transcript cleavage, while only three miRNAs perform the function of inhibiting target gene cleavage.

### 3.6 Expression profiling of *PeCNL* genes under multiple stresses

**FIGURE 10:**
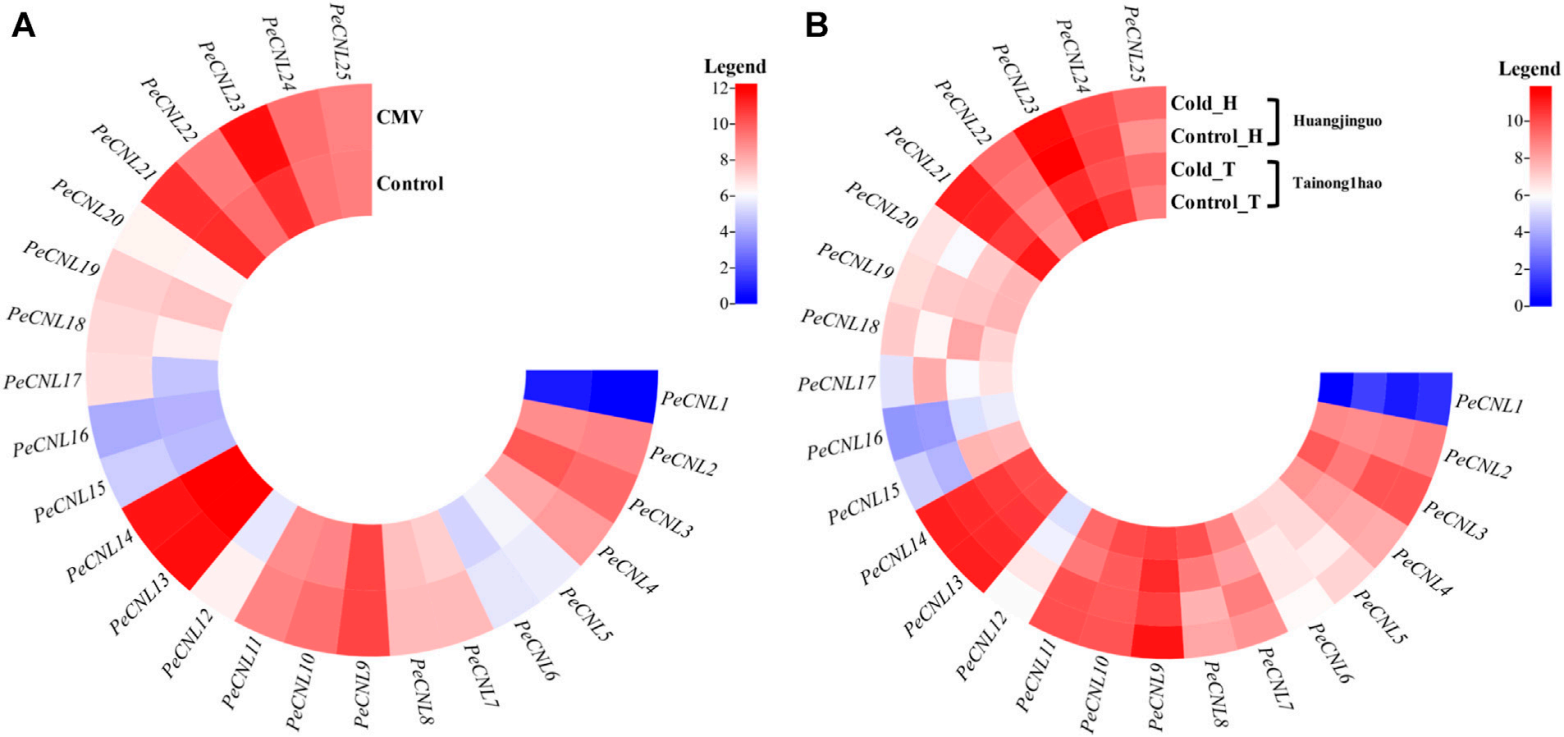
**(A)** The heatmap illustrates the expression levels of *PeCNL* genes under cucumber mosaic virus (CMV). **(B)** The heatmap depicts the expression levels of *PeCNL* genes by providing cold condition to the two cultivars of *P. edulis* Sims. namely, Tainong1hao and Huangjinguo. In the heatmap, dark cyan color indicates downregulated genes, white color represents no change in expression and red color signifies upregulated genes. The scale for the heatmap represents the log2 transformed count values.

The expression patterns were validated for 25 *PeCNL* genes of *P. edulis* which was subjected to CMV infection and cold condition. The heatmap represented that *PeCNL2*, *PeCNL4*, *PeCNL6*, *PeCNL7*, *PeCNL8*, *PeCNL10*, *PeCNL11*, *PeCNL12*, *PeCNL17*, *PeCNL18*, *PeCNL23* and *PeCNL24* were upregulated and *PeCNL3*, *PeCNL5*, *PeCNL13*, *PeCNL14*, *PeCNL16*, *PeCNL21*, and *PeCNL25* were downregulated under CMV infection (Figure 10A; Supplementary Table S6). The *PeCNL9* had no change in expression level under the CMV condition. Under cold condition *PeCNL3*, *PeCNL4*, *PeCNL6*, *PeCNL7*, *PeCNL16*, *PeCNL17*, *PeCNL19*, *PeCNL23*, and *PeCNL24* were slightly downregulated in both cultivars. While *PeCNL9*, *PeCNL13*, *PeCNL14*, *PeCNL15*, *PeCNL18*, *PeCNL22*, and *PeCNL25* were upregulated under the cold conditions in both cultivars (Figure 10B). Whereas, gene expression levels for remaining *PeCNL* genes were variable i.e., upregulated in one cultivar and downregulated in the second cultivar. The genes that are differentially expressed were the potential targets of *in-vitro* studies in the future after experimental validation and these include *PeCNL3*, *PeCNL13*, *PeCNL14*, *PeCNL23*, and *PeCNL21*.

### 3.7 Validation of *PeCNLs* under multiple stresses via machine learning

A total of 3 common genes namely, *PeCNL3*, *PeCNL13*, and *PeCNL14* were found to be differentially expressed that satisfied the criteria. All the 3 genes were upregulated in CMV infected condition while these were downregulated in cold condition. These genes are potentially significant to be used for making stress-resistant *P. edulis* Sims. varieties. These genes were used to test the performance of the Random Forest classifier already trained on CMV infected condition. *PeCNL3* yielded the best performance in terms of Accuracy, sensitivity, specificity, and AUC visualization (Supplementary Figure S1). Validating the expression of *PeCNLs* via machine learning would help explore the genes that are particularly responsible for multi-stress responsiveness. This can be used to improve *P. edulis* cultivar varieties soon which would have increased chances of survival by withstanding multiple stress conditions.

### 3.8 3D structure prediction and GO enrichment analysis of PeCNL proteins

**FIGURE 11:**
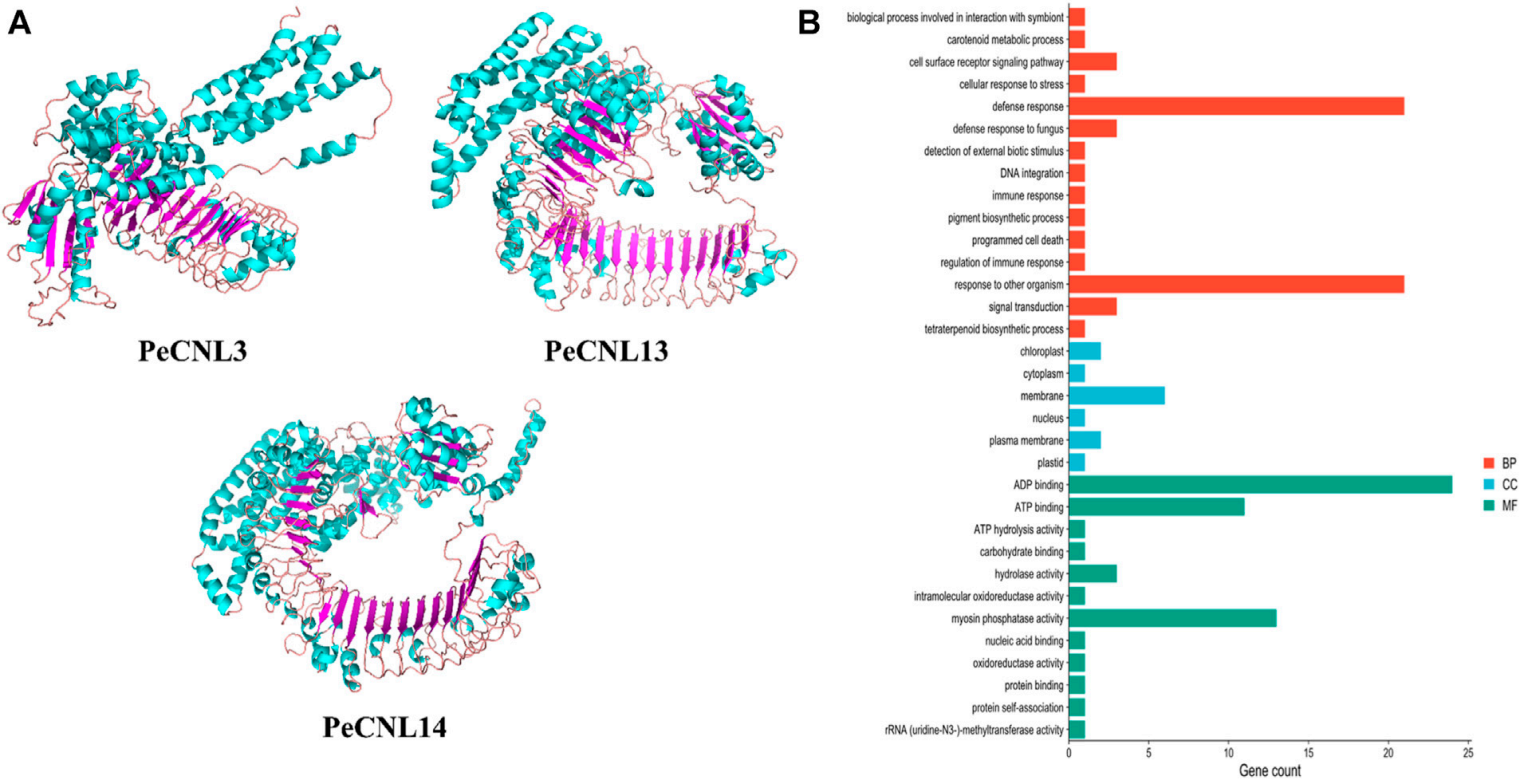
**(A)** 3D structures of the three PeCNL proteins predicted by trRosseta. Cyan color represents the alpha helices, purple color represents beta sheets, and light pink color represents the loops. **(B)** GO enrichment analysis of PeCNL proteins determined by using Pannzer2.

Based on the machine learning evaluation, three-dimensional structures were predicted for 3 PeCNL proteins namely, PeCNL3, PeCNL13, and PeCNL14 that were responsible for multi-stress responsiveness. PeCNL3 was found to have 35 alpha helices and 22 beta sheets, and PeCNL13 comprised 46 alpha helices and 29 beta sheets. While PeCNL14 contained 53 alpha helices and 30 beta sheets (Figure 11A). The variability in the number of alpha helices and beta sheets suggest that proteins might have undergone structural and functional divergence during the process of evolution to manage the survival of plant under changing conditions and pathogenic attack.

The GO enrichment analysis demonstrated the potential functions, biological processes, and cellular components in which each of the *PeCNL* proteins was involved. The majority of the *PeCNL* proteins were involved in ADP binding, ATP binding, and myosin phosphatase activity. Fewer proteins tend to be involved in other processes including ATP hydrolysis, hydrolase activity, and carbohydrate-binding activity. Accordingly, most of the *PeCNL* proteins were predicted to be present in the membranous part of the cell as already confirmed by subcellular localization. Others were located in the cytoplasm, nuclear, plastid, plasma membrane, and chloroplast sections. The GO enrichment analysis confirmed the involvement of PeCNL genes in the defense mechanism of *P. edulis* Sims. towards a variety of pathogens and environmental stresses (Figure 11B; Supplementary Table S7).

## 4 Discussion

The present work has utilized the most recent genome assembly of *P. edulis* Sims. and *P. edulis f. flavicarpa* and identified 25 *PeCNL* genes in *P. edulis* Sims. and 21 *PeCNL* genes in *P. edulis f. flavicarpa.* The identified *PeCNL* genes are smaller than those in *A. thaliana* (56) (Meyers et al., 2003), *Secale cereale (581)* (Qian et al., 2021)*, Glycine max (188)* (Nepal and Benson, 2015)*, Discorea rotundata (166)* (Zhang et al., 2020)*, C. sativus (33)* (Zhang et al., 2022)*, B. rapa (40)* (Liu et al., 2021)*, Oryza sativa L. var. Nipponbare (159)* (Zhou et al., 2004). The disparity in the number of identified CNL genes among other crops provides compelling evidence that this variation is a result of gene duplications or gene contraction events that likely occurred during evolution. The NLRs are immune receptors integral to the mechanism of ETI in plants. These receptors function as cytoplasmic proteins, responsible for discerning strain-specific effectors originating from pathogens. The localization of PeCNL proteins in cytoplasm and membrane gives evidence for the involvement of these proteins being a crucial part of the signaling pathway in targeting effectors released by pathogens.

The CNLs have been reported to be present in both monocots and dicots (Jacob et al., 2013) *RCY1* (Sekine et al., 2008)*, HRT* (Takahashi et al., 2002)*, RPP8/RPP13* (Bittner-eddy et al., 2000)*, RPM1* (El Kasmi et al., 2017)*, RPS2* (Ilag et al., 2000)*,* and *RPS5* (Qi et al., 2012) are *Arabidopsis AtCNL* genes that are validated through *in vitro* methods to be involved in conferring disease resistance in *A. thaliana* against various diseases including CMV, Turnip crinkle virus, Downy mildew of cucurbits, bacterial blight. However, the CNL genes in other plants have also been found to be validated experimentally for conferring disease resistance including five CNLs in *Solanum lycopersicum*, seven CNLs in *Triticum aestivum*, three CNLs in *Hordeum vulgare*, and eleven in *O. sativa* (Zhang et al., 2019). All of these findings suggest that as CNL genes have been proven to be involved in conferring disease resistance in other plants they will also be involved in conferring disease resistance in passion fruit. The characteristics of *PeCNLs* were consistent with the characteristics of *CsCNL* proteins (Zhang et al., 2022)*,* where the majority of the proteins were acidic only 13 proteins were basic, and the majority were localized to cytoplasmic and nuclear sections. The BrCNL proteins (Liu et al., 2021) were also similar to the PeCNL members because the majority were acidic.

Phylogeny inference based on the NJ method allowed the analysis of how PeCNL proteins linked to other proteins in the course of evolution. AtCNL proteins were divided into four groups in the phylogenetic tree namely, groups A, B, C, and D where the clade for group B was largest with 26 AtCNL members, and the clade for group A was smallest with 6 members. A comprehensive analysis incorporating the Viridiplantae kingdom in an already reported study unveiled that the genes encoding plant NLR proteins emerged from a shared ancestor of green plants and subsequently underwent divergent evolution, giving rise to three distinct subclasses during the early stages of plant evolution (Shao et al., 2019). All the PeCNL proteins were found to have close evolutionary relationships with CNL proteins of *A. thaliana* and *M. domestica*. The tree was divided into four groups namely, groups A, B, C, and D with a varied number of members in each group. Surprisingly none of the member of *P. edulis* Sims. was present in group D which was quite similar to the trends observed for CNL proteins of *C. sativus.* Thus, it can be inferred that AtCNLs and MdCNLs tend to be the orthologs of PeCNL proteins which indicates that they share the same ancestor.

All of the conserved motifs linked with the proper functioning of the *PeCNL* genes were found to be conserved in PeCNL proteins namely, P-loop, GLPL, Kinase-2, RNBS-B, RNBS-D, (Shao et al., 2016). The group C accompanied some PeCNL proteins that lack Kinase-2 motif and other motifs of unknown function. The motifs account for structural conformation of PeCNL proteins. The AtCNL proteins contained RNBS-A, RNBS-C, RNBS-D, and MHDV in addition to other conserved motifs (Meyers et al., 2003). *G. max,* contained seven conserved motifs (RNBS-A and RNBS-C) and along with other CNL specific motifs (Nepal and Benson, 2015). Exactly same set of conserved motifs were present in *Secale cereale* as in *P. edulis* Sims. (Qian et al., 2021)*.* Predicted conserved motifs in *D. rotundata* were same except for RSNB-D and P-loop (Zhang et al., 2020). However, the *C. sativus* contained additional motifs that were conserved in CsCNL proteins. In *B. rapa* the motifs 1, 5, 8, and 9 were responsible for unknown functions while other motifs were encoding NB-ARC and LRR domains. It can be concluded that the motifs are highly conserved across other plants because they are important for maintaining the structure and function of CNL proteins. The variability in motifs could be because each plant has undergone different environmental and selection pressures in the process of evolution.

Most of the *PeCNL* genes (11) had only one exon which represent 44% of the identified genes and 6 *PeCNL* genes had 3 exons that represent 24% of the total *PeCNL* genes. *PeCNL8* had 9 exons and 8 introns. Group A had exons ranging from 2 to 3 and introns ranging from 1 to 2. Group B had exons ranging from 1 to 3 and introns ranging from 1 to 2. Whereas, Group C had exons ranging from 1 to 9 and introns ranging from 1 to 4, and only one gene had 8 introns. In *C. sativus* (Zhang et al., 2022) Group A had 1, 3, and 5 exons respectively. Group B had exons in the range of 1 to 7. Group C had exons in the range of 1 to 5 with 1 being the most frequent. Among *BrCNL* genes Group I had 1, 2, and 11 exons respectively with 1 being the most frequent. Group II had an exon range given as 1 to 2. Group III had exons ranging from 1 to 3. Group IV had exons ranging from 1 to 5. Group V had exons ranging from 1 to 4. *AtCNL* genes and their gene products were encoded by single exons (Meyers et al., 2003). The number of introns impacts the expression speed of genes, so genes with a smaller number of introns can be faster edited and translated (Yaghobi and Heidari, 2023; Zaman et al., 2023). The differences in the number of exons and introns indicate the diversity in genic and intergenic regions of CNL genes in other plants and the variability in a number of gene family members in each plant.

All the *PeCNL* genes were distributed unevenly at 7 chromosomes and were present in the form of clusters. The CNL gene family being a subclass of the NLR gene family also tends genes to be clustered together likewise in the case of NLR where the size of these clusters varies considerably, with certain species possessing large clusters that include over 10 NLRs (Cesari et al., 2013). *PeCNL* genes formed the largest gene cluster at chromosome 3 with 7 genes representing 28% of the identified genes. Amongst the identified *PeCNL* genes none of them was present at chromosomes 6 and 7 which could be possibly due to gene contraction or gene transposition or due to the impact of environmental factors. In the case of *A. thaliana,* a total of 56 *AtCNL* genes were also distributed in the form of gene clusters at the five chromosomes. Based on a 10 ORF sliding window approach 41 gene clusters have been identified in the genome of *G. max.* Chromosome 10 did not contain any of the CNL gene and 105 genes (56%) were present at 5 out of 20 chromosomes (Nepal and Benson, 2015). Out of 582 *CNL* genes identified in the genome of *S. cereale* 111 *ScCNL* genes were present at chromosome 4 and almost half of these genes were present at chromosome 2 (Qian et al., 2021). The largest gene cluster of 22 genes was present at chromosome 3 of *D. rotundata* and the smallest gene cluster was at chromosome 21 with 3 genes (Zhang et al., 2020). The *BrCNL* genes formed a gene cluster at chr-A09 with 11 genes and the second largest cluster of genes was at chr-A06 with 8 genes respectively. *BrCNL* genes were completely absent at chr-A04 and chr-A07 and only one gene at chr-A02. Interestingly, *CsCNL* genes formed the largest gene cluster at chr2 with 10 genes. All these findings suggest the conservation of presence of CNL gene clusters on chromosomes across species.

A total of 34 duplicated gene pairs were found with an equal number of duplication events for both segmental and tandem duplicates, leading to the conclusion that both these duplication events contributed to expansions of the CNL gene family in *P. edulis* Sims*.* All of the duplicated gene pairs underwent strong purifying selection except *PeCNL8/PeCNL18* which was the product of positive selection. In *A. thaliana* a total of 149 *NLR* genes have been identified including CNL, TNL, and other subgroups. Out of the identified 149 *NLR* genes, 124 genes were the segmental duplication products indicating the association of gene duplication with the expansion of *CNL, TNL,* and other subgroups (Meyers et al., 2003). All of the identified CNL genes in *G. max* were the products of tandem duplication (Nepal and Benson, 2015). The dispersed, tandem and segmental duplications collectively accounted for the expansion of the *CNL* gene family in *S. cereale* with the dispersed playing the major counterpart (i.e., 60%) than the other two (i.e., 39% for tandem and 1% for segmental) (Qian et al., 2021). A total of 18 segmentally duplicated genes were found to be present in *D. rotundata* (Zhang et al., 2020). The Ka/Ks analysis of the NLR gene family in *Lagneria siceraria* (Wang et al., 2022) revealed that among 14 duplicated gene pairs, two gene pairs were segmentally duplicated, and the remaining were tandemly duplicated indicating that the tandem duplication was more favorable and all the duplicated gene pairs were products of negative selection. Ka/Ks analysis of the NLR gene family in *C. sinensis* (Yin et al., 2023) demonstrated that 16 duplicated gene pairs were tandemly duplicated and were a product of negative selection. The segmental and tandem duplications are equally contributing to the expansion of CNL gene family across the different plants.

The *cis-*elements were linked to growth and development, hormone response, and stress response. The majority of the *cis*-elements were involved in growth and development in comparison with CNL genes in *B. rapa* which contained mostly *cis-*elements for disease resistance. WBOX was the potential *cis*-element predicted to be present in the promoter regions of *G. max* thereby, regulating the defense-associated activity of *CNL* genes (Nepal and Benson, 2015). The hormone-related *cis-*elements are also responsible for pathogen-induced immune response where salicylic acid, Jasmonic acid (JA), and ethylene (ET), trigger signal transduction to activate PTI (Corina Vlot et al., 2009; Robert-Seilaniantz et al., 2011). In rice AS-1, G-box, GCC-box, and H-box are the potential *cis*-elements that induce pathogen defense (Kong et al., 2018). Similarly, *Brassica juncea* also has *cis*-elements associated with pathogen defense in the abiotic, biotic, and hormone related categories (Ali et al., 2017). By applying salicylic acid and Jasmonic acid treatment to plants the resistance of plants to pathogen attack gets increased or they promulgate pathogen-induced immune response (Argueso et al., 2012). The *cis*-element reported to confer pathogen resistance in *A. thaliana* include MYB, MYC, WRE3, W-box, STRE, and ARE (Saidi et al., 2024). The protein-protein interactions were found for the disease-resistance proteins of *A. thaliana* with PeCNLs. The TIR and PeCNL24 had a high degree of connectivity indicating that their function will be important for the survival of the plant in disease-related mechanisms.

The miRNAs are usually 18-20 nucleotides long and are responsible for regulating the function of PeCNL proteins. A total of 15 miRNAs targeted 19 *PeCNL* genes that further gained significant importance due to the way they regulate the functions of these proteins. The miRNAs offer a useful way for future disease management by targeting appropriate miRNAs.

The expression profiling of *PeCNL* genes was validated under CMV infection and cold stress. The *PeCNL3, PeCNL13,* and *PeCNL14* have differentially expressed genes under CMV and cold stress condition. *PeCNL3* and *PeCNL14* were downregulated under CMV condition and upregulated under cold condition. Contrastingly, *PeCNL13* was upregulated under CMV condition and downregulated in cold condition. *PeCNL3, PeCNL13*, and *PeCNL14* genes can withstand multiple stresses in *P. edulis* Sims. thus, suitable for developing stress-tolerant varieties of *P. edulis* Sims.

The expression patterns of *PeCNL* genes have been demonstrated under multiple stresses to find out the potential genes that are responsible for multi-stress responsiveness and useful for the defense mechanism of *P. edulis* Sims. to accommodate the underlying conditions. *PeCNL3, PeCNL13,* and *PeCNL14* were differentially expressed under multiple stresses. Machine learning approach i.e., the Random Forest model for regression has been applied to validate the expression of genes potentially involved in multi-stress responsiveness. *PeCNL3, PeCNL13,* and *PeCNL14* were found to be having a significant role in the multi-stress responsiveness of *PeCNL* genes thus indicating that they can be utilized as potential targets for making transgenic *P. edulis* varieties. The expression patterns of *CsCNL* genes were observed in seven tissues i.e., leaf, stem, root, male flower, female flower, tendril, and ovary, and abiotic and biotic stresses including Powdery mildew, downy mildew, salt stress, and low-temperature stress at different stages (Yin et al., 2023). The heatmaps demonstrated that most of the *CsCNL* genes have their expression level upregulated under abiotic and biotic stresses leading to the conclusion that these are involved in abiotic and biotic stresses and only a few genes were not exhibiting any change in their expression levels (Zhang et al., 2022b). 3D structures were predicted for the aforementioned proteins i.e., PeCNL3, PeCNL13, and PeCNL14. The number of alpha helices and beta sheets varied for each protein. The GO analysis confirmed the involvement of the PeCNLs in the mechanism of disease resistance. Thus, the identification of *PeCNL* genes in the genome of *P. edulis* would be crucial for gaining insights into how the *P. edulis* genome has expanded or evolved in the course of evolution to cope with changing environments and pathogens. Based on our analysis, it can be concluded that CNL genes could play a significant role in improving the genetic makeup of Passion fruit. These genes can be incorporated into breeding or genetic manipulation programs to provide disease resistance and enhance tolerance to abiotic stresses. Furthermore, the multi-stress responsiveness of these genes makes them valuable candidates for further breeding programs seeking to develop mango varieties that are adaptable to diverse environmental conditions. By breeding for PeCNL gene-related traits, we could achieve healthier plants, reduced pesticide dependency, and improved sustainability in Passion fruit cultivation.

## 5 Conclusion

In this study, a total of 25 and 21 *CNL* genes were identified in *P. edulis* Sims*. and Passiflora edulis f. flavicarpa,* respectively. The *PeCNL* genes were validated by the presence of conserved domains and motifs associated with the function of CNL genes. Phylogenetic analysis classified PeCNLs into four groups. Gene structure was highly conserved across *P. edulis* and other plants. Most of the PeCNL genes were present on chromosomes in the form of clusters. Both segmental and tandem duplications have been involved in the expansion of the CNL gene family in *P. edulis* Sims. *Cis-*regulatory elements were also found to be involved in growth and development, defense and stress, and hormone response of *PeCNL* genes. All of the PeCNL proteins were interacting with defense-related proteins. miRNA target prediction showed the regulatory roles in the expression of the PeCNL proteins. The varied number of alpha helices and beta sheets were present in PeCNL proteins and GO enrichment analysis confirmed the involvement of PeCNL proteins in the defense of plants against pathogens. The *PeCNL3, PeCNL13,* and *PeCNL14* were multi-stress responsive genes and were validated using machine learning approaches. Thus, the aforementioned genes could be crucial for the survival of plants underlying changing environmental conditions and pathogenic stress. After experimental validation, these genes could be increasingly helpful in making stress-tolerant varieties of *P. edulis* in the future.

## Data Availability

The original contributions presented in the study are included in the article/Supplementary Material, further inquiries can be directed to the corresponding author.
