# Supplementary figures and images for: Comparative genomics and bioinformatics approaches revealed the role of CC-NBS-LRR genes under multiple stresses in passion fruit
Source: Front Genet. 2024 Feb 26;15:1358134. doi: 10.3389/fgene.2024.1358134 (PMC10929019; doi:10.3389/fgene.2024.1358134)

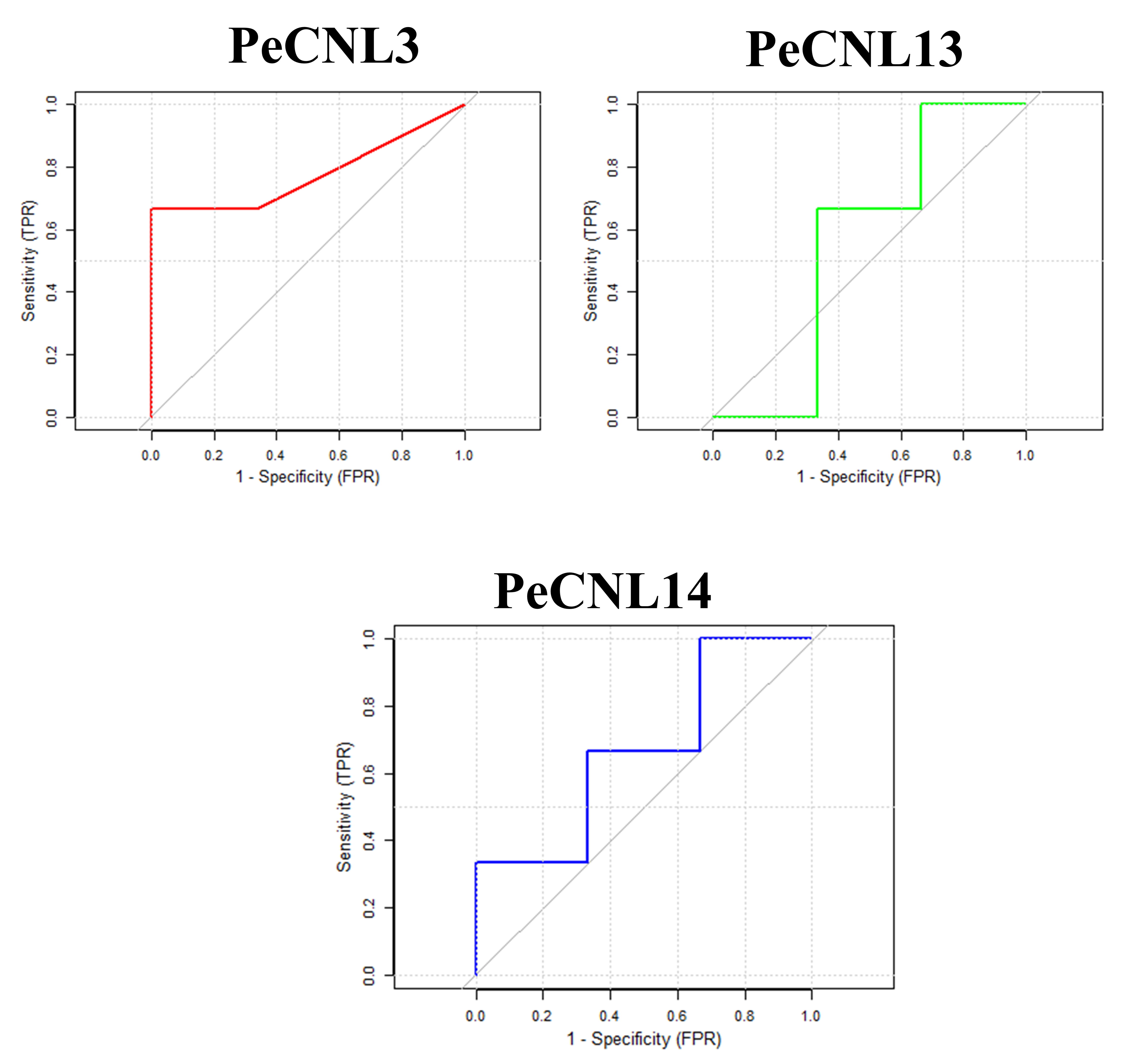

Supplement: Supplementary file 3 [file Image1.TIF]
